# Histological and immunohistochemical approaches to molecular subtyping in muscle-invasive bladder cancer

**DOI:** 10.3389/fonc.2025.1546160

**Published:** 2025-07-15

**Authors:** Giulio Attanasio, Maria Failla, Simone Poidomani, Tindaro Buzzanca, Serena Salzano, Maurizio Zizzo, Andrea Palicelli, Magda Zanelli, Nektarios Koufopoulos, Giorgio Ivan Russo, Rosario Caltabiano, Giuseppe Broggi

**Affiliations:** ^1^ Department of Medical, Surgical Sciences and Advanced Technologies “G.F. Ingrassia”, Anatomic Pathology, University of Catania, Catania, Italy; ^2^ Surgical Oncology Unit, Azienda USL-IRCCS di Reggio Emilia, Reggio, Emilia, Italy; ^3^ Pathology Unit, Azienda Sanitaria Locale-IRCCS di Reggio Emilia, Reggio Emilia, Italy; ^4^ Second Department of Pathology, Medical School, National and Kapodistrian University of Athens, Attikon University Hospital, Athens, Greece; ^5^ Urology Section, University of Catania, Catania, Italy

**Keywords:** muscle-invasive, molecular subtypes, immunohistochemistry, diagnosis, bladder cancer

## Abstract

Muscle-invasive bladder cancer (MIBC) is an aggressive form of bladder cancer, representing 20–25% of all bladder cancer cases. Characterized by invasion into the detrusor muscle, MIBC often leads to high rates of metastasis and poor outcomes, with five-year survival rates below 50% for localized disease and less than 15% for metastatic cases. MIBC primarily affects older adults, especially men, with smoking and chemical exposure being the leading risk factors. Clinically, MIBC presents significant heterogeneity, both histologically and molecularly, making diagnosis and management challenging. Histological variants of MIBC, such as squamous, micropapillary, plasmacytoid, and neuroendocrine subtypes, are associated with distinct prognoses and variable treatment responses. Recent advances in genomic profiling have identified molecular subtypes of MIBC—luminal, basal/squamous, neuronal, and stroma-rich—each with unique biological characteristics and treatment sensitivities. Despite these advancements, the widespread adoption of molecular profiling is hindered by the high costs and limited availability of these technologies, particularly in resource-limited settings. As a result, there is an increasing need for alternative, more accessible diagnostic methods to predict molecular subtypes. In this context, histological examination combined with immunohistochemical markers, such as GATA3, KRT5/6, and p63, has been shown to reliably correlate with molecular subtypes and guide therapeutic decisions. This review presents a comprehensive analysis of how histology, immunohistochemistry and molecular subtyping can be integrated into routine clinical practice to inform treatment strategies for MIBC, providing a pathway toward more personalized and effective management.

## 1 Introduction

### 1.1 Overview of muscle-invasive bladder cancer

Muscle-invasive bladder cancer (MIBC) is a particularly aggressive form of bladder cancer, characterized by its invasion into the detrusor muscle layer of the bladder wall. MIBC accounts for approximately 20-25% of bladder cancer cases at diagnosis and is distinct from non-muscle-invasive bladder cancer (NMIBC), which remains confined to the bladder’s superficial layers (1). While NMIBC has a more favorable prognosis, MIBC often presents a high risk of local invasion and metastasis. With an estimated 550,000 new cases and approximately 200,000 deaths annually, bladder cancer ranks as the 10th most common malignancy globally. MIBC represents a significant clinical challenge due to its aggressive nature and high recurrence and progression rates (2).

The incidence of bladder cancer is notably higher in men than women, with a male-to-female ratio of approximately 3:1. The median age at diagnosis typically falls between 65–70 years, with incidence rates increasing with age (2, 3). The overall five-year survival rate for MIBC remains below 50% for patients with localized disease, dropping to less than 15% in cases of distant metastasis. Radical cystectomy, often combined with neoadjuvant chemotherapy (NAC), is the standard treatment for MIBC; however, disease recurrence and progression are common even with aggressive therapy (4).

Smoking remains the predominant modifiable risk factor for bladder cancer, responsible for up to 50% of cases. Smoking exposes the bladder to carcinogens filtered from the bloodstream, substantially increasing cancer risk (5). Other major risk factors include occupational exposure to carcinogens, such as aromatic amines in industries like dye production and rubber manufacturing, chronic urinary tract infections, and previous pelvic radiation therapy (6).

The morbidity associated with MIBC is compounded by complications from radical cystectomy and systemic chemotherapy, which can affect quality of life due to urinary diversion, sexual dysfunction, and chemotherapy’s adverse effects. In addition, the high rates of recurrence and metastasis contribute to the disease’s overall mortality, with many patients ultimately succumbing to the disease despite initially curative treatments (7).

Non-modifiable risk factors include advanced age, male gender, and chronic inflammatory conditions such as chronic urinary tract infections or schistosomiasis in specific regions. Genetic predisposition also plays a significant role, as mutations in genes such as TP53 and RB1 are associated with more aggressive bladder cancer forms (8).

### 1.2 Aggressive nature and clinical presentation

MIBC is highly aggressive, marked by its propensity to invade nearby tissues such as the prostate, uterus, and pelvic walls, and metastasize to distant organs like the liver, lungs, and bones. This invasive behavior is facilitated by the tumor’s ability to infiltrate the detrusor muscle, allowing cancer cells to access lymphatic and vascular systems, which accelerates dissemination and complicates treatment (1, 9).

Early detection is crucial due to the rapid progression of MIBC. Unlike non-muscle-invasive bladder cancer (NMIBC), which can often be managed with localized treatments like transurethral resection (TURBT) or intravesical therapy, MIBC requires more aggressive interventions, such as radical cystectomy, frequently combined with NAC and immunotherapy (10). While outcome was associated with the consensus class for NAC-free patients, for NAC-treated patients, we observed no significant association of outcome with the consensus class (11). However, early detection of MIBC is often delayed because its symptoms can be non-specific and are easily mistaken for benign urinary conditions (12).

Clinically, MIBC often presents with symptoms indistinguishable from other common urological conditions, such as urinary tract infections (UTIs) or benign prostatic hyperplasia (BPH), especially in men. The most frequent symptom is hematuria, which can be either gross (visible to the naked eye) or microscopic (detectable only through laboratory tests). Hematuria in bladder cancer is typically painless, which often leads patients to delay seeking medical attention. Studies indicate that approximately 85% of bladder cancer patients experience hematuria at some stage of their disease (13).

In addition to hematuria, other urinary symptoms are common but are often mistaken for benign conditions. Patients may experience dysuria, characterized by painful or burning sensations during urination, as well as an increased frequency of urination or a constant sense of urgency, which may not be proportional to the amount of urine passed. Some patients also report nocturia, waking up multiple times at night to urinate. These symptoms can be subtle and progress gradually, leading both patients and physicians to attribute them to less serious conditions, further delaying diagnosis (13–15).

Once diagnosed, MIBC tends to progress rapidly, with a significant likelihood of recurrence or metastasis if left untreated or if treatment is delayed. Approximately 50% of patients with MIBC will eventually develop metastatic disease, even after aggressive local therapy, due to the early occurrence of micrometastasis during the disease’s progression (1, 16, 17).

The rapid progression of MIBC underscores the importance of early and accurate diagnosis to improve patient outcomes. Diagnosis is typically confirmed through cystoscopy, which allows for direct visualization of the bladder, and is followed by a biopsy to confirm histopathological characteristics. Imaging studies, such as Computed Tomography (CT) or Magnetic Resonance Imaging (MRI), are used to stage the disease and assess the extent of local invasion or distant metastasis (1, 18).

In recent years, molecular subtyping has gained importance in guiding personalized treatment strategies for MIBC. Molecular subtyping allows clinicians to classify tumors based on specific gene expression profiles, providing insight into the biological behavior of the cancer and its likely response to different treatment modalities. Integrating molecular subtyping with traditional histopathological analysis enhances diagnostic accuracy and enables patient stratification according to their risk of recurrence and metastasis. This approach ensures that patients with high-risk subtypes receive the most aggressive treatments early in their disease course, which is critical for improving survival outcomes (19–21).

## 2 Materials and methods

### 2.1 Search strategy

A thorough electronic literature search up to November 2024 was made, and the following databases were used to identify relevant articles: PubMed/MEDLINE, Cochrane Library (Cochrane Database of Systematic Reviews, Cochrane Central Register of Controlled Trials—CENTRAL), Web of Science (Science and Social Science Citation Index), and Scopus. The combination of non-MeSH/MeSH terms was as follows: (i) PubMed/MEDLINE: ((Muscle-Invasive Bladder Cancer [Title/abstract]) OR (Muscle-Invasive Bladder Urothelial Carcinoma [Title/abstract]) AND (Immunohistochemistry [Title/abstract])) OR (Molecular Subtypes [Title/abstract])). Filters applied: English; (ii) Cochrane Library: Muscle-Invasive Bladder Cancer in Title Abstract Keyword AND Molecular Subtypes in Title Abstract Keyword- (word variations have been searched). Language: English; (iii) Web of Science: Muscle-Invasive Bladder Cancer (Topic) AND Immunohistochemistry (Topic) OR Molecular Subtypes (Topic) and English (Languages); and (iv) Scopus: (TITLE-ABS-KEY (Muscle-Invasive Bladder Cancer) AND TITLE-ABS-KEY (Immunohistochemistry) OR TITLE-ABS-KEY (Molecular Subtypes) AND (LIMIT-TO (LANGUAGE, “English”)). Additionally, the reference lists of relevant studies were manually reviewed to identify any articles that may have been missed during the electronic search.

### 2.2 Inclusion and exclusion criteria

Articles not addressing Muscle-Invasive Bladder Cancer or those discussing it from a perspective other than the immunohistochemical and molecular subtypes were excluded. Comments, opinions, perspectives, guidelines, editorials, case reports, and papers in languages other than English were also excluded. In addition, papers available only as abstracts or those with text appearing too brief or non-informative were not included into the present narrative review. The inclusion criteria focused on articles in English with full texts available. We concentrated on articles that offered thorough summaries or in-depth discussions of histological and molecular subtypes of Muscle-Invasive Bladder Cancer and their applications in prognosis, and clinical management.

## 3 Results

Fourteen histological variants of MIBC were identified, each with distinct morphological features, prognostic significance, and therapeutic implications. Conventional urothelial carcinoma (NOS) remains the most common subtype, while aggressive variants such as plasmacytoid, sarcomatoid, and micropapillary carcinomas are associated with worse outcomes and limited responsiveness to standard therapies. Variants like squamous and glandular differentiation exhibit divergent behavior, necessitating tailored therapeutic strategies. Rare forms including clear cell, lipid-rich, and trophoblastic variants were also described, each contributing to the complexity and clinical challenge of MIBC management. The review outlines four consensus molecular subtypes—Luminal, Basal/Squamous, Neuronal, and Stroma-Rich—each defined by distinct genetic and transcriptional profiles. Luminal subtypes are characterized by GATA3, KRT20, and UPK2 expression and demonstrate responsiveness to FGFR inhibitors. Basal/Squamous subtypes exhibit aggressive behavior but respond favorably to platinum-based neoadjuvant chemotherapy, with key markers including KRT5/6, KRT14, and p63. Neuronal subtypes express neuroendocrine markers such as SOX2 and synaptophysin and require small-cell lung cancer–like chemotherapy regimens. Stroma-rich tumors, marked by mesenchymal and immune markers (e.g., vimentin, PD-L1), are often chemoresistant but may benefit from immunotherapeutic strategies. Immunohistochemistry (IHC) has emerged as a practical and cost-effective surrogate for molecular profiling in settings where genomic technologies are limited. Panels based on GATA3, KRT5/6, p63, and neuroendocrine markers enable approximation of molecular subtypes and stratification of patients for appropriate treatment pathways. The integration of histology with IHC enhances subtype classification and supports more personalized clinical decision-making, particularly in resource-constrained environments.

All relevant findings were extracted and arranged in a narrative manner.

## 4 Histological subtypes of muscle-invasive bladder cancer

MIBC represents a highly heterogeneous disease, with substantial variability at both histological and molecular levels. This heterogeneity manifests through distinct histological subtypes and molecular profiles, each with unique biological behaviors and clinical implications. Understanding these differences is critical for optimizing treatment strategies, as the various subtypes profoundly affect prognosis, therapeutic decision-making, and overall clinical outcomes (10, 22, 23).

Histologically, MIBC is predominantly classified as urothelial carcinoma, also known as transitional cell carcinoma. However, there are less common but aggressive histological variants, including squamous differentiation, micropapillary carcinoma, plasmacytoid carcinoma, and neuroendocrine carcinoma, each displaying unique histopathological features and distinct clinical behaviors that often correlate with poorer outcomes (24) (**Table 1**). Understanding MIBC heterogeneity is crucial for developing targeted treatment strategies and personalized care plans, since these variants can impact treatment selection and prognosis, presenting therapeutic challenges due to their aggressive nature and resistance to standard chemotherapy regimens (11, 25, 26).

**Table 1:** Histologic subtypes of muscle-invasive bladder cancer.

Histologic Subtype	Characteristics	Prognosis	Key immunoistoche mical markers	Clinical Implications
*Urothelial Carcinoma (NOS)*	High pleomorphism, papillary or solid growth	Poor if muscle- invasive	None specific	Good response to neoadjuvant chemotherapy; Radical cystectomy, NAC (Neoadjuvant Chemotherapy)
*Squamous Differentiation*	Squamous cells, often in response to chronic irritation	Poor	p63, CK5/6	Limited response to cisplatin; Radiation therapy, immunotherapy in clinical trials
*Micropapillary*	Floating cell nests resembling lymphovascular invasion, high early metastatic potential	Poor	HER2	Limited response; Anti-HER2 therapies like trastuzumab (under study)
*Plasmacytoid*	Plasma cell-like cells with higher tendency for peritoneal dissemination	Poor	CD138, loss of E- cadherin (CDH1)	Low chemotherapy response; Immunotherapy and targeted therapies under research
*Nested*	Benign appearance but deeply invasive	Variable to Poor	None specific	Limited based on stage; Cystectomy for invasive forms
*Lymphoepitheliom a-Like*	Pleomorphic cells within dense lymphoid infiltrate, resembling nasopharyngeal carcinoma	Good	CD8+, PD-L1	Good response to platinum-based chemo; Platinum-based chemotherapy
*Clear Cell (Glycogen-Rich)*	Glycogen-rich cytoplasm of clear cells	Poor	PAS-Diastase positive	Low chemotherapy response; Early surgical intervention
*Lipid-Rich*	Lipid vacuoles in tumor cells, highly pleomorphic	Poor	None specific	Low chemotherapy response; Aggressive treatment needed
*Giant Cell*	Multinucleated giant cells, marked cellular atypia	Poor	None specific	Advanced stage, poor prognosis
*Neuroendocrine I*	Small, round ndocrine	Poor	Synaptophysin, hron.og in	Responsive to etoposide-cisplatin chemo; therapy with PD-L1 inhibitors
*Sarcomatoid*	M enchymal non, presillice of osteosarcomatous or chondrosarcomatous elements	Poor	Vimentin	Cystectomy, poor prognosis
*Microcystic*	Small cystic spaces, resembling cystitis cystica, highly invasive	Good	None specific	Cystectomy for deep invasion
*Glandular Differentiation*	Gland-like differentiation, mucin production	Poor	None specific	Limited; Intensive systemic therapy
*Trophoblastic Differentiation*	Resembles placental syncytiotrophoblast cells, with hCG production	Poor	hCG	Poor; Aggressive treatment required
*Poorly Differentiated*	Extreme pleomorphism, loss of typical architecture	Poor	None specific	Poor, early metastatic tendency; Multimodal treatment approaches

### 4.1 Urothelial carcinoma (Not Otherwise Specified)

**Figure 1:**
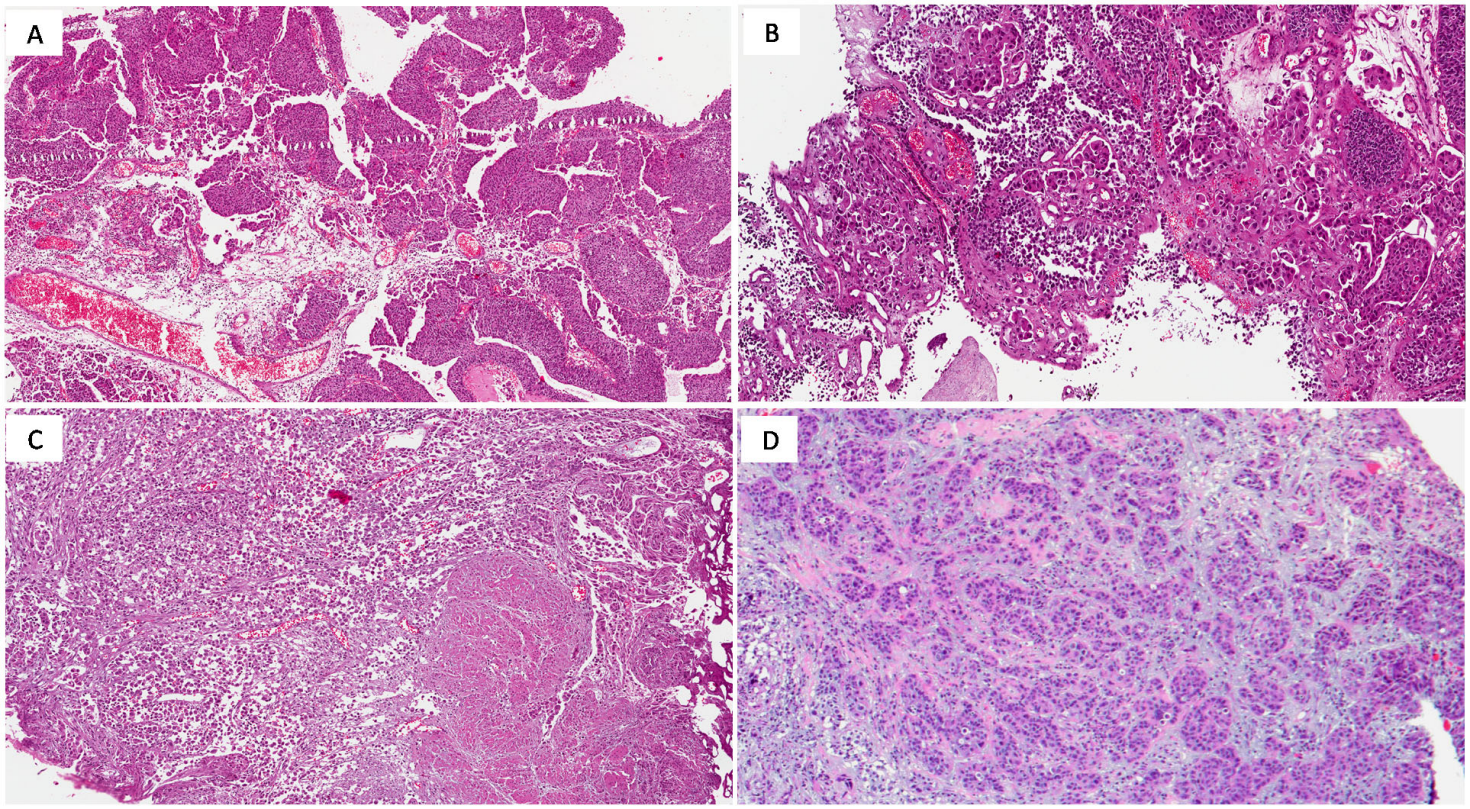
**(A)** High-grade urothelial carcinoma (NOS) showing papillary growth-pattern (H&E; original magnification 150x); **(B)** Micropapillary urothelial carcinoma (H&E; original magnification 150x); **(C)** Plasmacytoid urothelial carcinoma exhibiting deep infiltration into the muscularis propria (H&E; original magnification 150x); **(D)** Nested urothelial carcinoma (H&E; original magnification 150x).

Urothelial carcinoma, Not Otherwise Specified (NOS) accounts for approximately 80-90% of MIBC cases and originates from the transitional epithelium lining the bladder. The term NOS is used for cases that do not exhibit specific histological variants or other molecular characteristics. This subtype can present in various architectural patterns, including papillary, solid, or nested forms. The hallmark of muscle invasive urothelial carcinoma is muscularis propria infiltration, indicative of advanced disease (**Figure 1A**) (26). Histological features such as high-grade nuclear pleomorphism, hyperchromasia, and extensive necrosis contribute to its poor prognosis. For high-grade, muscle-invasive tumors, radical cystectomy combined with NAC is often the preferred treatment (27).

### 4.2 Squamous differentiation

Squamous differentiation is observed in about 20% of urothelial carcinoma cases, typically arising in response to chronic irritation, such as from bladder stones, recurrent infections, or schistosomiasis (28). Histologically, it is recognized by keratinization and intercellular bridges. Squamous differentiation is associated with a more aggressive clinical course and poor responsiveness to cisplatin-based chemotherapy, necessitating alternative treatments like radiation or immunotherapy (29).

### 4.3 Micropapillary urothelial carcinoma

Micropapillary urothelialcarcinoma (MPUC) is a rare yet highly aggressive variant of MIBC, often diagnosed at advanced stages due to its early tendency to metastasize. Microscopically, it is characterized by small, cohesive tumor cell nests that mimic lymphovascular invasion (**Figure 1B**). This distinct appearance correlates with an aggressive clinical course and poor prognosis, thus radical cystectomy is frequently recommended. Adverse outcomes despite aggressive platinum-based chemotherapy and limited response to Bacillus-Calmette-Guerin instillation therapy have been reported in MPUC, but little is known about alternative treatment strategies and most authors recommend early aggressive surgical treatment (27, 30–32). HER2 overexpression is common in micropapillary carcinoma and represents a potential therapeutic target, although the use of HER2-targeted therapies like trastuzumab is still under clinical investigation (33).

### 4.4 Plasmacytoid urothelial carcinoma

Plasmacytoid carcinoma is an aggressive variant of MIBC, accounting for approximately 1-3% of cases. It is often diagnosed at an advanced stage and is characterized by widespread infiltration and peritoneal dissemination. Histologically, plasmacytoid carcinoma is marked by discohesive tumor cells resembling plasma cells, featuring eccentric nuclei and eosinophilic cytoplasm (**Figure 1C**). These tumors frequently display a solid, sheet-like growth pattern, making surgical resection challenging.

CDH1 mutations, which result in the loss of E-cadherin, are commonly observed in this variant, contributing to its discohesive nature and aggressive behavior. CD138 (syndecan-1) positivity further supports the plasmacytoid differentiation by highlighting the plasma cell-like features of these tumors. Advanced cases often show a desmoplastic stromal response, correlating with a worse prognosis (34). Due to its aggressive behavior and poor response to chemotherapy, ongoing research is exploring targeted therapies and immunotherapy as potential treatment options (35).

### 4.5 Nested urothelial carcinoma

Nested urothelial carcinoma is a rare variant recognized for its deceptively bland appearance, which can complicate differentiation from benign proliferations like von Brunn nests. It presents in small and large nested forms, with the small nested variant being more common (**Figure 1D**). The subtype is marked by nests of urothelial cells with minimal atypia, especially in superficial layers (36, 37).

However, deep infiltration into the muscularis propria is a key diagnostic feature signaling malignancy. Despite its benign appearance, nested carcinoma has a poor prognosis due to deep tissue invasion and delayed diagnosis (37, 38).

### 4.6 Lymphoepithelioma-like urothelial carcinoma

Lymphoepithelioma-like carcinoma, a rare MIBC subtype, bears a strong resemblance to nasopharyngeal lymphoepithelioma. This variant occurs as either a pure form or alongside conventional urothelial carcinoma. It is characterized histologically by large pleomorphic cells with prominent nucleoli, often surrounded by a dense inflammatory infiltrate of lymphocytes and plasma cells (39). Despite its aggressive appearance, the pure form often has a favorable prognosis and responds well to platinum-based chemotherapy. When coexisting with conventional carcinoma, prognosis depends on the non-lymphoepithelioma component (39, 40).

### 4.7 Clear cell (glycogen-rich) urothelial carcinoma

Clear cell carcinoma is a rare MIBC variant featuring glycogen-rich, clear cytoplasm, often resembling renal clear cell carcinoma. Histologically, it is characterized by cells with abundant glycogen-rich cytoplasm, identifiable through special staining techniques like periodic acid-Schiff (PAS) with diastase digestion (41). Although the clinical course of clear cell carcinoma is not well documented due to its rarity, it generally has a poor prognosis. Early surgical intervention remains the primary treatment approach (42).

### 4.8 Lipid-rich urothelial carcinoma

Lipid-rich carcinoma is a rare and aggressive subtype of MIBC, marked by the presence of lipid vacuoles within tumor cells. These vacuoles compress the nuclei, giving cells a lipoblast-like appearance. This variant is highly pleomorphic, with significant nuclear atypia and mitotic activity, and is associated with poor prognosis due to extensive local invasion (43, 44). Early recognition and aggressive treatment are crucial, though outcomes remain poor given its resistance to conventional therapies (45).

### 4.9 Sarcomatoid urothelial carcinoma

**Figure 2:**
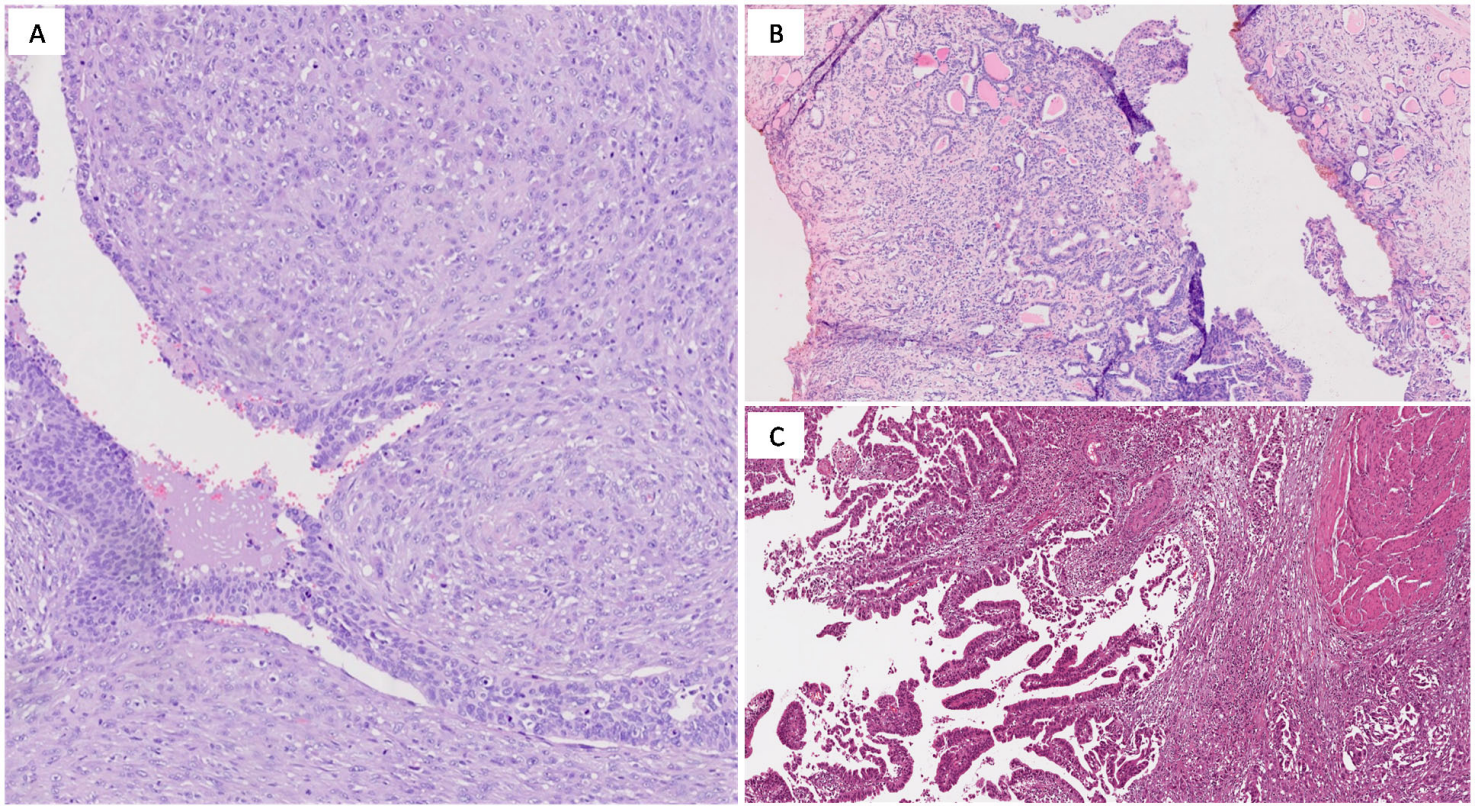
**(A)** Sarcomatoid urothelial carcinoma showing spindle cell sarcomatous differentiation (H&E; original magnification 200x); **(B)** Microcystic urothelial carcinoma consisting of bland- looking tubules lined by urothelium (H&E; original magnification 150x); **(C)** Muscle-invasive urothelial carcinoma with glandular differentiation, resembling intestinal-type adenocarcinoma (H&E; original magnification 150x).

Sarcomatoid carcinoma is an aggressive variant exhibiting both epithelial and mesenchymal differentiation, with high-grade spindle or pleomorphic cells (**Figure 2A**) and occasional heterologous elements like osteosarcoma or chondrosarcoma (46). This subtype frequently presents with metastasis at diagnosis and is associated with a worse prognosis than conventional urothelial carcinoma (47). Radical cystectomy is commonly recommended, though the aggressive nature of the disease often results in poor outcomes (48).

### 4.10 Microcystic urothelial carcinoma

Microcystic carcinoma is a rare form of MIBC that can be mistaken for benign conditions like cystitis cystica (49). It features small cystic or tubular spaces lined by urothelial cells (**Figure 2B**), which can delay diagnosis due to its benign appearance (50). Despite this morphology, the tumor often invades deeply, leading to poorer prognosis. Radical cystectomy is usually required to manage the aggressive nature of this subtype (51).

### 4.11 Urothelial carcinoma with glandular differentiation

Glandular differentiation, the second most common form of divergent differentiation in urothelial carcinoma, is characterized by gland-like structures and enteric differentiation with glandular formations (**Figure 2C**) and mucin production (52). It can resemble colonic adenocarcinoma or mucinous adenocarcinoma, and sometimes contains signet-ring cells. This variant often correlates with a more aggressive course and higher metastasis risk, necessitating intensive treatment (52).

### 4.12 Urothelial carcinoma with trophoblastic differentiation

This exceedingly rare variant resembles placental syncytiotrophoblast cells and is marked by Human Chorionic Gonadotropin (hCG) production. Histologically, it includes multinucleated giant cells, with elevated hCG serving as a tumor marker. Trophoblastic differentiation is associated with a particularly poor prognosis and usually requires radical treatment approaches due to its aggressive nature (53).

### 4.13 Giant cell urothelial carcinoma

One of the most aggressive bladder cancer forms, giant cell carcinoma is defined by multinucleated giant cells and significant cellular atypia. The presence of large, irregular cells and extensive necrosis reflects a highly aggressive and rapidly progressing tumor (54). Patients often present at advanced stages, and prognosis is generally poor even with aggressive interventions like radical cystectomy (54, 55).

### 4.14 Poorly differentiated urothelial carcinoma

This subtype is characterized by extreme nuclear pleomorphism, irregular nuclei, and frequent mitoses. Poorly differentiated tumors lack cohesive growth patterns and have an aggressive clinical course with early metastasis and deep tissue invasion. Treatment typically involves aggressive multimodal approaches, though outcomes remain guarded (56, 57).

## 5 Molecular subtypes of muscle-invasive bladder cancer

The molecular classification of muscle-invasive bladder cancer (MIBC) has significantly advanced our understanding of the disease’s complexity, providing a foundation for predicting treatment responses and clinical outcomes. Widely accepted subtypes—Luminal, Basal/Squamous, Neuronal, and Stroma-Rich—arise from major classification systems such as The Cancer Genome Atlas (TCGA) and the Consensus Molecular Subtypes (CMS), which integrate genetic, epigenetic, and expression data to create a cohesive framework for diagnosis and treatment selection. Beyond these core categories, evolving subtypes like luminal immune-high and claudin-low underscore the expanding molecular heterogeneity recognized within MIBC, offering additional dimensions for guiding therapeutic strategies. Several alternative molecular subtyping approaches further enrich this landscape; for example, the TCGA classification delves into complex multi-omic profiles, enabling precise therapeutic targeting but requiring substantial resources, while the University of North Carolina (UNC) classifier simplifies MIBC into essential luminal and basal subtypes, prioritizing clinical practicality with slightly less granularity. RNA-based signature classifiers provide another promising method, using gene expression data to predict responses to chemotherapy and immunotherapy, though these approaches demand RNA-sequencing infrastructure, which may limit accessibility. Together, these subtyping methodologies deepen our understanding of MIBC, allowing clinicians to tailor strategies based on available resources, patient needs, and specific tumor biology, ultimately advancing personalized care in bladder cancer (58).

### 5.1 Luminal subtype

The luminal subtype is one of the most common molecular categories in MIBC, marked by characteristics associated with urothelial differentiation and signaling pathways that resemble those found in normal urothelial cells. This subtype is generally associated with a better prognosis, showing lower rates of recurrence and metastasis compared to more aggressive subtypes (59).

Driven by molecular alterations that promote urothelial differentiation, the luminal subtype features markers such as GATA3 and KRT20, which are essential transcription factors and cytokeratins involved in luminal epithelial cell differentiation (60). FGFR3 mutations, primarily activating mutations, are frequently observed in this subtype, enhancing urothelial differentiation and making these tumors candidates for FGFR inhibitors. One of the most significant advancements for patients with FGFR3-mutated luminal tumors is the development of erdafitinib, an FGFR inhibitor that has shown efficacy in clinical trials and is now an FDA-approved therapy for this mutation (61).

HER2/ERBB2 overexpression is also observed in some luminal tumors, particularly in the luminal-papillary subtype. This has opened the door to the exploration of HER2-targeted therapies, such as trastuzumab, which has shown promise in early trials for patients with HER2-positive bladder cancer (62). Although trastuzumab is widely used in breast cancer, ongoing studies are evaluating its role in improving outcomes for luminal MIBC with HER2 overexpression (62).

The responsiveness of luminal tumors to NAC remains an area of active investigation, as these tumors tend to be less sensitive to traditional chemotherapy than the basal subtype. For example, luminal-papillary tumors, which often harbor FGFR3 mutations, are less responsive to NAC and show better outcomes with FGFR-targeted therapies (61). In addition, low response rates for immune checkpoint inhibitors have been found in basal squamous subtype and luminal infiltrated subtype, which had the highest levels of immune infiltration (63).

### 5.2 Basal/squamous subtype

The basal/squamous subtype is characterized by aggressive tumor biology, with molecular features reminiscent of basal cells found in the skin or breast. It is defined by the expression of basal cell markers KRT5/6, KRT14, and p63, which are involved in maintaining the stem-cell-like properties of tumor cells (10). These markers suggest a more primitive, undifferentiated cell origin, contributing to the aggressive clinical behavior of these tumors. This subtype shows worse overall survival than the luminal subtype but exhibits better responsiveness to platinum-based chemotherapy regimens.

Patients with basal/squamous MIBC often show TP53 and RB1 mutations, which are typically inactivating; these mutations contribute to genomic instability and enhance chemosensitivity to neoadjuvant chemotherapy by reducing the tumor’s ability to repair DNA damage effectively (10). However, the prognosis remains poor due to high rates of recurrence and metastasis after treatment.

The plasmacytoid variant, notable for its discohesive growth pattern and frequent loss of E-cadherin due to CDH1 mutations, is often included within the basal/squamous category because of its similarly aggressive nature and poor prognosis (34, 35).

Emerging research has shown that basal tumors might particularly benefit from immunotherapy, especially with immune checkpoint inhibitors. For example, atezolizumab, a PD-L1 inhibitor, has been shown to be effective in MIBC cases expressing PD-L1, offering a promising therapeutic approach in basal subtypes (64).

The basal subtype frequently expresses EGFR and sometimes HER2, making it a candidate for EGFR-targeted therapies. Lapatinib, a dual EGFR/HER2 inhibitor, is being explored for its potential in treating basal MIBC, especially in tumors with HER2 overexpression or EGFR activation (64).

Although lapatinib is more commonly used in breast cancer, its application in bladder cancer, particularly for EGFR-positive basal subtypes, is being investigated in clinical trials (64). Another promising area of research for basal MIBC involves STAT3 inhibitors. These drugs target the STAT3 signaling pathway, which is involved in tumor progression and immune evasion. Although STAT3 inhibitors are still experimental and not yet available as standard treatment, early clinical studies show potential for these inhibitors in basal tumors that rely on the STAT3 pathway (65).

Thus, the combination of platinum-based chemotherapy, immune checkpoint inhibitors, and targeted therapies like EGFR inhibitors offers a multifaceted approach to treating the aggressive basal/squamous subtype of MIBC (64, 65).

### 5.3 Neuronal subtype

The neuronal subtype is the most aggressive molecular subtype of MIBC, exhibiting molecular and clinical characteristics similar to neuroendocrine tumors. This subtype is typically associated with the worst prognosis among all molecular classifications and necessitates highly specialized treatment approaches (58, 66). Neuronal subtype tumors express markers associated with neuroendocrine differentiation, such as SOX2, synaptophysin, and chromogranin A, which are involved in neurogenesis regulation and are commonly observed in small-cell neuroendocrine carcinomas.

Genomic studies reveal frequent alterations in TP53, RB1, and MYC pathways, driving the rapid proliferation and aggressive behavior of these tumors (58, 59).

The neuronal subtype is highly sensitive to etoposide-cisplatin (EP) chemotherapy, a regimen often utilized for treating small-cell lung cancer. This chemotherapy approach has shown efficacy in controlling tumor growth in neuronal MIBC; however, despite aggressive treatment, the prognosis remains poor due to the high likelihood of early metastasis (59, 67). Emerging research is also evaluating immune checkpoint inhibitors for this subtype, but the heavily altered genome suggests variable immune responsiveness (10). While rare, neuroendocrine bladder cancers exhibit high sensitivity to immunotherapy due to unique molecular features, including elevated PD-L1 expression.

Consequently, patients with neuroendocrine tumors may benefit from customized immunotherapy regimens, potentially combined with chemotherapy, given their aggressive behavior and rapid progression (68, 69).

Among the MIBC molecular subtypes, the neuronal subtype has the worst prognosis, characterized by high mortality rates and poor overall survival. Most patients present with advanced-stage disease, and long-term survival remains limited even with aggressive chemotherapy. Early identification and treatment are crucial to prolong survival; however, the outlook for patients remains challenging (68–70).

### 5.4 Stroma-rich subtype

The stroma-rich subtype of MIBC is distinguished by a significant presence of stromal or mesenchymal components within the tumor microenvironment. This subtype represents a unique molecular category, influenced heavily by interactions between tumor cells and the surrounding stroma. Stroma-rich tumors exhibit high expression of mesenchymal markers, such as vimentin and smooth muscle actin, indicating epithelial-to-mesenchymal transition (EMT)—a process in which epithelial cells acquire fibroblast-like properties that enhance invasiveness and metastatic potential (58, 59). In addition to EMT markers, these tumors also express elevated levels of fibroblast activation markers and stromal-related genes, underscoring the crucial role of the stromal microenvironment in driving tumor behavior (71).

Stroma-rich tumors are often resistant to standard chemotherapy, particularly platinum-based regimens. he dense stroma can act as a physical barrier, impeding drug penetration, while the mesenchymal signaling pathways active in these tumors contribute to intrinsic chemoresistance (26). However, the stromal component also suggests a potential sensitivity to immune checkpoint inhibitors. Research is ongoing to explore how the tumor stroma modulates immune responses, with a focus on therapies targeting the PD-1/PD-L1 axis that may be effective in such cases (72).

The dense stromal content in stroma-rich tumors, characterized by a high abundance of fibroblasts and other stromal cells, poses significant challenges for immunotherapy. The stroma can serve as a physical and immunosuppressive barrier, restricting the infiltration of immune cells, particularly T cells. High levels of transforming growth factor-beta (TGF-β) signaling in these tumors further promote an immunosuppressive environment, often confining immune cells to the periphery of the tumor and reducing the efficacy of immune checkpoint inhibitors (73–85). Consequently, combination therapies that target both stromal components and enhance immune cell infiltration are being actively investigated. For instance, clinical trials are assessing the efficacy of combining TGF-β inhibitors with checkpoint inhibitors to break down stromal barriers and facilitate immune cell access to the tumor (62, 76).

The prognosis for stroma-rich tumors can be variable, but these tumors are generally associated with higher resistance to conventional therapies and an increased propensity for metastasis. Nonetheless, the potential for immunotherapy opens new therapeutic avenues, particularly for patients who have not responded to traditional chemotherapy (76, 77).

## 6 Immunohistochemical markers for molecular subtyping

The use of IHC markers in the molecular subtyping of MIBC is critical for diagnostic pathology and therapeutic decision-making. IHC offers a reliable and cost-effective method for identifying molecular subtypes, particularly in clinical settings where comprehensive genomic profiling may not be accessible. By targeting specific protein expressions, IHC enables the classification of MIBC into subtypes such as Luminal, Basal, Neuronal, and Stroma/Immune-rich (**Table 2**). These subtypes have significant clinical implications, especially in predicting treatment response and survival outcomes (78).

**Table 2:** Molecular subtypes of muscle-invasive bladder cancer.

Molecular Subtype	Characteristics	Prognosis	Therapeutic Implications
*Luminal*	Urothelial differentiation, FGFR3 mutations; markers include GATA3, KRT20.	Generally better; lower recurrence and metastasis.	Potential candidates for FGFR inhibitors; less responsive to NAC.
*Basal/Squamou s*	Basal cell markers (KRT5/6, KRT14); TP53 and RB1 mutations common	Poorer overall survival, higher recurrence and metastasis rates.	Good response to platinum-based NAC; research into DNA damage repair inhibitors.
*Neuronal*	Neuroendocrine differentiation, SOX2, synaptophysin, chromogranin	Worst prognosis, high mortality and early metastasis.	Sensitive to etoposide-cisplatin chemotherapy; exploring immune checkpoint inhibitors.
*Stroma-Rich*	High stromal content, EMT markers (vimentin); mesenchymal transition	Variable prognosis, often chemotherapy-resistant	Potential for immunotherapy; PD- 1/PD-L1 inhibitors being investigated.

### 6.1 Luminal markers

The luminal subtype of MIBC is predominantly characterized by markers that indicate urothelial differentiation. Essential IHC markers for identifying luminal tumors include GATA3, Cytokeratin 20 (CK20), and Uroplakin 2 (UPK2). While HER2 is more commonly associated with aggressive subtypes like micropapillary urothelial carcinoma, its role in standard luminal MIBC remains limited.

These markers are integral for both diagnosis and the development of targeted therapeutic strategies (58–60, 79, 80) (**Table 3**).

**Table 3:** Immunohistochemical markers for MIBC molecular subtype.

Molecular Subtype	Marker	Interpretation criteria
*Lioninal*	GATA-3	Moderate to strong nuclear staining
*Lioninal*	CK20	Moderate cytoplasmic staining
*Lioninal*	UPK2	Moderate to strong membranous staining
*Basal/Squanous*	CK5/6	Moderate to strong cytoplasmic staining
*Basal/Squanous*	CK14	Moderate cytoplasmic staining
*Basal/Squanous*	p63	Strong ruslear staining
*Basal/Squanous*	E-Cadherin and CD138	E-Cadherin absence of membranous staining and CD138 membranous positivity for plasmocytoid variant
*Basal/Squanous and Neuronal*	p53-mutated	Three patterns: (1) Strong nuclear accumulation, (2) Complete absence or "rull-phenotype", (3) cytoplasmic staining
*Neuronal*	Synaptophysin	Strong cytoplasmic staining
*Neuronal*	Chromogranin	Strong cytoplasmic staining
*Neuronal*	SOX2	Moderate to strong nuclear staining
*Stroma- Rich/Imprache-Rich*	PD-L1	Tumor Proportion Score (TPS ≥1-5%) and Combined Positive Score (CPS ≥10); membranous staining in tumor and immune cells
*Stroma-Rich*	Vimentin	Moderate to strong cytoplasmic staining
*Stroma-Rich*	CD8	Moderate to strong cytoplasmic staining
*Stroma-Rich*	FOXP3	Moderate to strong nuclear staining

GATA3 is a transcription factor that plays a critical role in the differentiation of urothelial cells. It is one of the most sensitive and specific markers for luminal bladder cancer, displaying nuclear staining in the majority of cases. Expression of GATA3 is observed in approximately 90% of luminal tumors, reliably indicating this subtype (78). Its nuclear staining pattern helps distinguish luminal tumors from other subtypes. KRT20 is an epithelial marker associated with luminal cell differentiation and typically exhibits cytoplasmic staining. It is highly prevalent in luminal MIBC, making it a valuable marker alongside GATA3 for identifying luminal tumors. While KRT20 is commonly used as a marker, evidence supporting its role in predicting response to neoadjuvant chemotherapy is limited, and further studies are required to clarify its prognostic value (81). UPK2, a transmembrane protein integral to the urothelial umbrella cell layer, presents a membranous staining pattern in luminal bladder cancer. This marker is highly specific to urothelial cells, strengthening the classification of tumors as luminal. UPK2, along with GATA3 and KRT20, provides a robust IHC panel to identify luminal tumors when molecular testing is unavailable or impractical (78, 81).

HER2 overexpression and/or amplification is observed more frequently in aggressive subtypes of bladder cancer, such as micropapillary urothelial carcinoma, which is known for its aggressive behavior and poor prognosis. HER2 assessment in bladder cancer is conducted similarly to breast cancer, utilizing IHC to evaluate protein overexpression and fluorescence *in situ* hybridization (FISH) for gene amplification. HER2-targeted therapies may benefit patients with micropapillary bladder cancer, but its relevance in standard luminal subtypes is less clear (82). In micropapillary cases, determining HER2 status can assist in selecting targeted treatments, potentially improving outcomes for affected patients (83).

### 6.2 Basal markers

The expression of basal markers, such as KRT5/6, KRT14, and p63, has been associated with an enhanced response to NAC, particularly with platinum-based regimens. However, recent research indicates that the response to NAC in basal tumors may also depend on other factors, such as the tumor’s molecular profile and the presence of immune markers like PD-L1. Basal tumors, which often express these markers, are more likely to achieve pathologic downstaging following NAC, leading to improved survival outcomes. IHC profiling can help identify patients most likely to benefit from NAC and more aggressive treatment approaches (59).

Cytokeratins 5 and 6 are critical basal cell markers that exhibit strong cytoplasmic staining in basal/squamous subtype tumors. Their expression highlights the stem cell-like traits of basal tumors, which are often poorly differentiated and more aggressive. KRT5/6 expression correlates with shorter overall survival, as these tumors are frequently diagnosed at advanced stages with squamous differentiation (59, 83).

KRT14 is another cytoplasmic basal cell marker that is frequently co-expressed with KRT5/6, reinforcing the basal/squamous characteristics of MIBC. This marker underscores the clinically aggressive nature of basal-like tumors. Additionally, KRT14 expression is associated with poor prognosis, particularly in tumors co-expressing EGFR and p53, which further emphasize the aggressive phenotype (59, 83).

In MIBC, p53 presents in one of three patterns: strong nuclear positivity, cytoplasmic accumulation, or a null phenotype. These patterns correspond to mutations that generally inactivate p53’s tumor-suppressive function, correlating with poor prognosis and resistance to cisplatin-based chemotherapy.

The null phenotype, indicative of complete loss of p53, is linked to severe genomic instability. These p53 patterns, often seen alongside basal markers like KRT5/6, help identify high-risk patients and support personalized treatment strategies. The presence of p53 mutations, particularly when associated with RAS pathway activation, is known to drive EMT in MIBC, promoting a basal phenotype and aggressive cancer cell behavior (84, 85).

The nuclear protein p63 is a crucial tumor suppressor in basal cells and is fundamental in identifying the basal subtype of bladder cancer. It maintains stem cell-like properties in epithelial cells and is frequently co-expressed with KRT5/6 and KRT14. The nuclear expression pattern of p63 aligns with the aggressive clinical behavior commonly associated with basal markers (58, 59).

### 6.3 Markers for neuronal subtype

Neuroendocrine tumors expressing markers like SOX2, Synaptophysin, and Chromogranin A generally exhibit high aggressiveness and poor prognosis. These markers help guide treatment, often involving EP chemotherapy regimens similar to those used in small-cell lung cancer due to shared neuroendocrine characteristics. However, emerging treatments such as immune checkpoint inhibitors (anti-PD-1/PD-L1) are showing promise in improving outcomes for patients with neuroendocrine bladder cancers, particularly those with high levels of T cell infiltration (86, 87). Early identification of these markers through IHC enables timely initiation of aggressive treatment, although long-term survival remains limited for patients with neuroendocrine bladder cancers (86, 87).

SOX2 is a transcription factor crucial for maintaining pluripotency in stem cells, frequently demonstrating nuclear staining in neuroendocrine tumors of the bladder. It is associated with aggressive clinical behavior and poor prognosis. In addition to promoting EMT, SOX2 has been implicated in therapeutic resistance and tumor plasticity, further enhancing tumor invasiveness (88–90).

Synaptophysin is a membrane glycoprotein that exhibits cytoplasmic staining and is a reliable indicator of neuroendocrine differentiation. Its presence in bladder cancer suggests a neuronal subtype, typically seen in more aggressive forms, such as small-cell neuroendocrine carcinomas.

Recently, the marker INSM1 has been identified as another promising marker for neuroendocrine tumors, potentially improving diagnostic accuracy when combined with synaptophysin and chromogranin A (91). Synaptophysin is instrumental in distinguishing neuroendocrine bladder cancers from other subtypes, aiding in accurate diagnosis (91). Chromogranin A, displaying cytoplasmic staining, is a classic marker for neuroendocrine cells. It is often co-expressed with Synaptophysin, confirming the neuroendocrine subtype. Elevated levels of Chromogranin A are linked to poor prognosis and reduced survival rates in neuroendocrine bladder cancer patients (91).

### 6.4 Stroma/immune markers

Stroma-rich tumors frequently show substantial resistance to chemotherapy due to the physical and biochemical barriers posed by their dense stromal components. However, the presence of markers like PD-L1 and CD8 suggests potential sensitivity to immune checkpoint inhibitors. This underscores the importance of early identification through IHC analysis, which can guide the selection of targeted therapies, particularly when conventional chemotherapy proves ineffective (89). These markers, particularly PD-L1 and CD8, are essential in selecting candidates for immunotherapy. However, recent studies indicate that PD-L1 expression is not always a definitive predictor of response, an responses to immune checkpoint inhibitors can occur even in patients with low PD-L1 expression.

Therefore, combining PD-L1 assessment with other markers, such as CD8 and tumor mutational burden (TMB), may provide a more reliable stratification method for immunotherapy candidates (89, 90).

PD-L1 is expressed on tumor and immune cells within the tumor microenvironment, facilitating immune evasion by the tumor. High PD-L1 expression is linked to better responses to immune checkpoint inhibitors, such as anti-PD-1/PD-L1 therapies. However, the correlation between PD-L1 levels and immunotherapy response is not absolute, and other factors, such as CD8+ T cell infiltration, TMB, and IFN-γ expression, are being increasingly used to predict response more accurately (90, 91).

CD8 is a marker for cytotoxic T cells, indicating an active immune response within the tumor. High infiltration of CD8+ cells is associated with improved prognostic outcomes and enhanced responses to immunotherapy, as these cells directly target and eliminate cancer cells. Studies show that increased CD8+ T cell presence correlates with favorable responses to treatments, including chemotherapy and immune checkpoint inhibitors. Furthermore, the ratio of CD8+ T cells to FOXP3+ regulatory T cells (Tregs) may serve as a more precise predictor of immunotherapy success than CD8+ cell infiltration alone (90, 91).

FOXP3 is a marker for Tregs, contributing to an immunosuppressive environment within the tumor.

High FOXP3 expression is typically associated with poor prognosis due to its role in immune suppression, which may facilitate tumor growth and resistance to therapies. However, the CD8+ T cell to FOXP3+ Treg ratio offers additional insight into the tumor’s immune landscape and can be a more accurate predictor of therapeutic response. Recent research indicates that Tregs can have dual roles, promoting immune suppression in some contexts but potentially beneficial in others by regulating chronic inflammation (91–93).

Vimentin is a key marker of mesenchymal cells and plays a critical role in EMT, which promotes tumor invasiveness. In stroma-rich MIBC, vimentin exhibits cytoplasmic staining and is associated with aggressive clinical behavior. Its expression highlights the stromal and mesenchymal characteristics of this subtype, suggesting a tumor environment conducive to increased invasiveness and metastatic potential. Additionally, EMT plasticity, including mesenchymal-to-epithelial transition (MET), is also involved in metastatic progression and could be a target for future therapeutic strategies (90, 91).

## 7 Diagnostic approach in the absence of molecular profiling

Molecular profiling methods, such as next-generation sequencing (NGS) and transcriptomic analysis, have significantly enhanced the diagnosis and treatment of MIBC. These technologies facilitate precise molecular subtyping, enabling personalized treatment strategies. In recent years, the cost of NGS and transcriptomic analyses has decreased, and their accessibility has improved in many clinical settings, including smaller hospitals and cancer centers. This has made these technologies increasingly available, reducing the limitations cited in the past due to high costs and the need for specialized infrastructure (26, 59). However, integrating histological examination with IHC markers still provides a cost-effective alternative that offers valuable insights into tumor biology and enables accurate subtyping without full molecular data (26, 59).

The diagnostic process starts with a detailed histological examination, identifying structural characteristics of the tumor. For instance, papillary architecture often signifies luminal subtypes, which tend to have a favorable prognosis but are less responsive to chemotherapy. Luminal tumors, frequently harboring FGFR3 mutations, may benefit from FGFR-targeted therapies. Recent studies reinforce the efficacy of Erdafitinib, an FDA-approved FGFR inhibitor for MIBC patients with FGFR3 mutations, providing an alternative to traditional chemotherapy for patients with luminal tumors. Additionally, innovative approaches are under investigation, such as bispecific FGFR3 antibodies that inhibit FGFR3 dimerization, which offer promise for patients resistant to tyrosine kinase inhibitors (TKIs) (94, 95).

IHC markers further refine subtype confirmation. In luminal MIBC, markers like GATA3, KRT20, and UPK2 confirm urothelial differentiation, supporting a diagnostic pathway that may lead to alternative therapies rather than chemotherapy. In settings where molecular profiling techniques are not available, immunohistochemistry offers a valuable approach to approximate molecular subtypes and guide prognostic and therapeutic stratification. Basal/squamous tumors express markers such as KRT5/6, KRT14, and p63, indicating basal cell traits. These tumors, despite a poorer prognosis, respond well to NAC, which is critical for treatment planning (28). For basal/squamous subtypes, characterized by squamous differentiation or basal-like aggressive features, neoadjuvant chemotherapy (NAC) remains a primary choice due to high chemosensitivity. Immunotherapy is also increasingly considered, given that the immune environment in these tumors often favors immune response. STAT3 inhibitors represent an additional therapeutic option for this subtype, as the STAT3 pathway is frequently active in basal tumors, where it promotes growth and immune evasion. STAT3 inhibitors like TTI-101 and SH5–07 have shown efficacy in reducing tumor proliferation and enhancing cell death, potentially augmenting responses to existing therapies. Another STAT3 inhibitor, WP1066, has demonstrated effectiveness in reducing tumor invasiveness and improving immune response, making it promising for reducing tumor spread (96, 97). For tumors exhibiting neuroendocrine characteristics, the neuronal subtype can be identified by markers like SOX2, synaptophysin, and chromogranin. These tumors are highly malignant and typically require aggressive chemotherapy akin to regimens for small-cell lung cancer. Tumors with significant stromal or immune cell infiltration fall into the stroma/immune-rich subtype, often expressing PD-L1 and CD8 markers, which highlight immune activity and suggest responsiveness to immune checkpoint inhibitors (91, 94). However, recent studies have shown that the effectiveness of immunotherapy is not solely dependent on PD-L1 expression, but also on other factors like T-cell cytosis and the TMB (91, 93). An algorithmic approach, incorporating histological and IHC findings, streamlines the subtyping process. Identifying histological features and using specific IHC markers help confirm the subtype and correlate with known clinical behaviors. For example, luminal tumors, with their susceptibility to FGFR mutations, may be better suited for FGFR inhibitors, whereas basal/squamous tumors necessitate aggressive treatments due to high chemotherapy responsiveness (59).

## 8 Limitations of histology and immunohistochemistry in muscle-invasive bladder cancer subtyping

Histology and IHC provide practical and accessible means of subtyping MIBC, yet they have limitations that can impact diagnostic accuracy and reliability. One primary challenge is intratumoral heterogeneity, as MIBC tumors often contain diverse cellular populations with varying molecular and histological traits. A biopsy sample may not fully capture this diversity, potentially leading to inaccurate molecular subtype identification. This sampling bias can affect treatment decisions, as the chosen therapy may not address the tumor’s full complexity. Recent advances in diagnostic methods, such as multiregional profiling and AI-assisted analysis, are being developed to mitigate these challenges (98, 99).

Additionally, there is variability in IHC staining protocols across different laboratories due to differences in antibody selection, staining conditions, and interpretation criteria. Such inconsistencies can lead to divergent IHC results, with the same tumor potentially classified into different subtypes depending on the facility. New international guidelines aim to standardize IHC protocols to reduce these discrepancies. However, despite these guidelines, standardization remains a challenge in diverse clinical settings, where access to specific antibodies and optimized staining protocols may be limited, potentially leading to inconsistency in subtype classification (96, 97). This variability highlights the ongoing need for standardized protocols to improve diagnostic consistency.

Inter-observer variability further complicates the interpretation of IHC results, as staining intensity and patterns are often subjective and heavily reliant on the pathologist’s expertise. This subjectivity introduces a significant source of inter-observer variability, where different pathologists may reach different conclusions based on the same data. To address this, AI-based tools are being integrated into pathology workflows to assist in standardizing interpretations and reducing subjectivity. Despite the promise of AI, challenges remain, as these tools still require extensive validation to ensure they can reliably replicate human judgment in diverse clinical scenarios. Such variability can significantly affect MIBC subtype classification and subsequent treatment choices (3, 100).

While markers such as GATA3 and CK20 are frequently utilized for identifying the luminal subgroup, and CK5/6 and CK14 for the basal subgroup, further validation is necessary to confirm their reliability in defining MIBC subgroups. Hybrid techniques combining IHC and molecular profiling have emerged as promising tools to enhance diagnostic accuracy by integrating histological features with genetic data. However, the integration of molecular data presents its own challenges, including the high costs associated with molecular profiling and the need for sophisticated infrastructure and technical expertise. Consequently, there remains a need for accessible yet accurate methods that can serve as practical alternatives to comprehensive molecular profiling, particularly in settings with limited resources (98). Finally, the reliance on IHC and histology for MIBC subtyping raises questions about bias in current methodologies, as these approaches may not capture the full spectrum of molecular heterogeneity present in tumors. Subtypes identified solely through IHC may overlook significant genetic and epigenetic variations, resulting in a less comprehensive understanding of tumor biology. As research continues to refine IHC panels and standardize protocols, there is a pressing need for more studies validating these markers across diverse patient populations and clinical settings to minimize diagnostic discrepancies and optimize the use of IHC in MIBC subtyping. The ongoing effort to improve histology and IHC subtyping seeks to bolster the overall diagnostic reliability for MIBC, while providing clinicians with robust tools for accurate subtyping to facilitate better-informed, personalized treatment decisions even in the absence of molecular profiling (3).

## 9 Future directions in muscle-invasive bladder cancer research and clinical applications

Future research in muscle-invasive bladder cancer (MIBC) should prioritize the validation of novel biomarkers to improve subtyping accuracy and reduce diagnostic variability, emphasizing multicenter studies for broad applicability. Integrating genomic data with histology and immunohistochemistry (IHC) can address the limitations of single-modality diagnostics, allowing a more complete characterization of tumor heterogeneity and enabling targeted therapy. The identification of new therapeutic targets, such as mutations in FGFR3 and HER2, remains critical, particularly for aggressive subtypes where innovative treatments are needed. Developing hybrid diagnostic protocols that incorporate histology, IHC, and molecular data could enhance accessibility while maintaining accuracy, especially in settings without extensive molecular testing capabilities.

Furthermore, AI and machine learning offer valuable tools for standardizing IHC interpretations and reducing observer bias, with potential to expand into real-time analysis that combines histological, IHC, and genomic information. Together, these advances aim to refine diagnostic precision, support personalized treatment decisions, and ultimately improve patient outcomes in MIBC management.

## 10 Conclusions

MIBC is not only histologically diverse, with subtypes like squamous differentiation and neuroendocrine variants, but also exhibits significant molecular heterogeneity, which adds complexity to its diagnosis and treatment (101–103).

Advancements in genomic profiling have led to the identification of specific molecular subtypes—namely luminal, basal/squamous, neuronal, and stroma-rich. Each of these subtypes presents unique biological behaviors and sensitivities to different treatments, making molecular subtyping invaluable for personalizing therapeutic strategies. However, the widespread adoption of molecular profiling is hindered by its high costs and limited accessibility, particularly in settings with fewer resources. This limitation underscores the necessity for alternative diagnostic methods that can approximate molecular subtypes without relying on genomic technologies.

Histopathology, combined with IHC markers, offers a feasible and cost-effective solution for predicting molecular subtypes in clinical practice. Key IHC markers, such as GATA3 for luminal subtypes and KRT5/6 and p63 for basal/squamous subtypes, enable clinicians to approximate the molecular characteristics of MIBC and tailor treatments accordingly. This approach not only broadens access to subtype-specific insights but also facilitates a more personalized approach to managing MIBC, especially where genomic profiling is not available.

Incorporating histology and IHC into routine practice allows healthcare providers to make informed decisions about patient care based on accessible and reliable data. As more research validates the effectiveness of these markers for predicting molecular subtypes, histological and IHC methods are likely to become even more integral to the management of MIBC. In summary, while molecular profiling remains the gold standard, histology and IHC present a viable alternative that can enhance the precision of treatment strategies, offering a practical pathway to improved patient outcomes in diverse clinical settings.
